# Effects of Tearing Conditions on the Crack Propagation in a Monolayer Graphene Sheet

**DOI:** 10.3390/ijms23126471

**Published:** 2022-06-09

**Authors:** Jiao Shi, Weihua Yu, Chunwei Hu, Haiyan Duan, Jiaxing Ji, Yuanyuan Kang, Kun Cai

**Affiliations:** 1 Key Laboratory of Agricultural Soil and Water Engineering in Arid and Semiarid Areas, Ministry of Education, Northwest A&F University, Yangling 712100, China; shijiaoau@163.com; 2 College of Water Resources and Architectural Engineering, Northwest A&F University, Yangling 712100, China; yuweihua@nwsuaf.edu.cn (W.Y.); huchunwei51@163.com (C.H.); jijiaxing1994@163.com (J.J.); 3 Material Science Department, School of Forestry, Northwest A&F University, Yangling 712100, China; yy_kang152@163.com; 4 School of Science, Harbin Institute of Technology, Shenzhen 518055, China

**Keywords:** graphene patterning, crack propagation, tearing load, molecular dynamics

## Abstract

The path of crack propagation in a graphene sheet is significant for graphene patterning via the tearing approach. In this study, we evaluate the fracture properties of pre-cracked graphene during the tearing process, with consideration of the effects of the aspect ratio, loading speed, loading direction, and ambient temperatures on the crack propagation in the monolayer sheet. Some remarkable conclusions are drawn based on the molecular dynamic simulation results, i.e., a higher loading speed may result in a complicated path of crack propagation, and the propagation of an armchair crack may be accompanied by *sp* carbon links at high temperatures. The reason for this is that the stronger thermal vibration reduces the load stress difference near the crack tip and, therefore, the crack tip can pass through the *sp* link. A crack propagates more easily along the zigzag direction than along the armchair direction. The out-of-plane tearing is more suitable than the in-plane tearing for graphene patterning. The path of crack propagation can be adjusted by changing the loading direction, e.g., a rectangular graphene ribbon can be produced by oblique tearing. This new understanding will benefit the application of graphene patterning via the tearing approach.

## 1. Introduction

Graphene [1] is a type of *sp*^2^-bonded carbon material exhibiting remarkable electronic [2,3], thermal [4], and mechanical [5,6,7] properties, meaning that the innovative low-dimensional material has wide applications [8,9,10,11,12,13,14,15,16,17]. When working as a functioning component of a nanodevice, a graphene ribbon/sheet needs to be patterned with specific geometry. Meanwhile, defects in the graphene pattern should be avoided to guarantee its mechanical robustness, which determines the service life of the device. For example, Bai et al. [11] fabricated a graphene nanomesh with lithography. The nanomesh behaved as a semiconductor in a field-effect transistor. Blees et al. [12] prepared a graphene kirigami that can act as a nanobalance. Yang et al. [13,14] investigated the thermal stability of nanoweaves from graphene ribbons. Cai et al. [15,16] built nanosprings and a nanospring-based network for energy storage using partially hydrogenated graphene ribbons. Shi et al. [17] introduced a graphene nanoflake as a rotor in a rotary nanomotor. Muniz et al. [18,19] developed twisted bilayer graphene with periodic *sp*^3^ interlayer bonds, and estimated its mechanical properties via molecular dynamics (MD) simulations. Cai et al. [20] introduced a graphene ribbon in a rotor to show the centrifugal force at the nano scale [21,22]. Song et al. [23,24] modelled a rotary nanodevice for achieving the transfer of rotation–translation. Cai et al. [25,26] built a rotary nanomotor with graphene origami as actuators. They [27] also suggested a way to weld a cut graphene ribbon into a cone. Obviously, fabricating the above graphene-based nanodevices should be based on the accurate patterning of a graphene sheet.

In preparing the graphene-based components in a nanodevice or nanostructure, several types of patterning techniques have been developed, e.g., lithographic techniques [19,28,29,30], irradiation methods [31], chemical etching [32,33,34,35,36], and mechanically controllable break junctions [1,37,38,39]. The tearing approach is one of the simplest mechanical approaches. In a tearing process, an initial crack is first introduced on the graphene sheet, and then the two edges beside the crack are stretched in opposite directions. In tearing, the fracture toughness of the graphene sheet describes the ability of the material containing a crack to resist fracture. Thus, the fracture behavior of graphene has attracted much attention in the last decade. For example, Huang et al. [40] discussed ways to control the tearing paths in graphene through chemical functionalization, using MD simulations. Kim et al. [41] investigated the crack formation and propagation mechanisms in suspended graphene sheets using transmission electron microscopy. They found that the edges from ripping exhibit straight lines, and are predominantly aligned in the armchair or zigzag directions. Omeltchenko et al. [42] performed MD simulations to investigate crack propagation in a monolayer graphene sheet, and observed that in the perpendicular orientation, multiple crack branches sprouted off the primary crack front at regular intervals. They also calculated fracture toughness in terms of the stress intensity factor of the graphene sheet to be 4.7 MPa·m^1/2^. Xu et al. [43] reported similar values, i.e., 4.21 MPa·m^1/2^ for zigzag and 3.71 MPa·m^1/2^ for armchair cracks. Moura and Marder [44] studied the propagation of cracks in graphene, and found that the path of crack propagation and the newly generated edge structure depend on the initial length of the crack. Khare et al. [45] estimated the energy-release rate at the point of crack extension in graphene by using coupled quantum mechanical/molecular mechanical modeling. Zhang et al. [46] designed a 3D graphene structure with controlled distributions of topological defects. The constructed graphene structure had a fracture toughness of around 25 J/m^2^, which is about twice that of pristine graphene. Budarapu et al. [47] studied the crack growth mechanisms and the variation in temperature during crack growth in a graphene sheet with an initial crack under uniaxial tension. Wang and Liu [48] investigated the fracture toughness of graphene with grain boundaries, and showed that these defects can block crack propagation and affect fracture toughness significantly in bi-crystal graphene under tensile force. The experimental results of Lopez-Polín et al. [49] also showed that while tears in pristine graphene span microns in length, crack propagation is strongly reduced in the presence of defects, suggesting controlled defect creation as an approach to avoid catastrophic failure in graphene.

**Figure 1:**
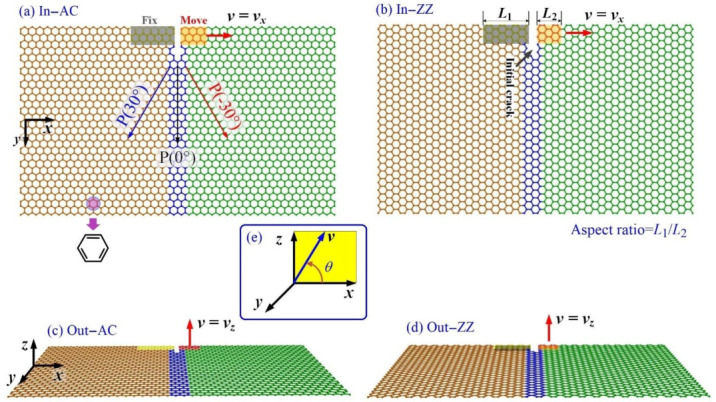
Geometry of monolayer graphene sheets with armchair (AC) crack or zigzag (ZZ) crack. The atoms in the fixed area with a length of *L*_1_ are fixed in tearing. Meanwhile, the atoms in the moving area with a length of *L*_2_ move synchronously along a direction with the speed of *v*. The ribbons have similar sizes of ~100.8 Å × ~60.3 Å. The depth of the initial crack is ~5 Å. (**a**) In-plane tearing along the armchair crack (In-AC), and (**b**) along the zigzag crack (In-ZZ); (**c**) out-of-plane tearing along the armchair crack (Out-AC), and (**d**) along the zigzag crack (Out-ZZ). (**e**) Oblique tearing. The vector ***v*** is in the xz-plane. “*θ* = 0°” means an in-plane tearing, while “*θ* = 90°” means an out-of-plane tearing. “0° < *θ* < 90°” illustrates an oblique tearing.

In the above studies, which have provided a deep understanding of graphene fractures, much attention has been paid to functionalization [40], types of defects [49,50,51], and the effects of the process of out-of-plane tearing. In an experiment, the essential factors—such as loading speed, ambient temperature, tearing direction, and the lengths of the confined areas on the target graphene for loading—also influence the tearing results, which have received less attention in previous studies. To enrich the understanding of fractures in graphene, in the present study, we focus on the influence of these factors on the tearing results using the MD simulation approach, with the tearing modes given in Figure 1.

In Figure 1, the length of the fixed area, i.e., *L*_1_, has three options. The length of the moving area, i.e., *L*_2_, has four options, and determines the value of the aspect ratio (AR = *L*_1_/*L*_2_). Correspondingly, the three pairs of models are referred to as AC1, ZZ1; AC2, ZZ2; and AC3, ZZ3, with the values of AR listed in Table 1. For visualization, the atoms in the central part of the graphene sheet are highlighted in blue. The speed of the moving area, i.e., *v*, is constant in the tearing process. The moving direction of the moving area determines the tearing mode, e.g., in-plane (Figure 1a,b), out-of-plane (Figure 1c,d), or oblique tearing (Figure 1e).

The tearing displacement is defined as the product of *v* and *t*, i.e., *d* = *v*·*t*. The path of crack propagation is formed when the C-C bonds at the crack tip break one by one. There are many possible paths, including the three paths—referred to as, P(0°), P(−30°), and P(30°)—marked in Figure 1a. For simplicity, a path can be defined by labeling the angle (rotating from 0° or the direction of the initial crack) in the bracket. For example, P(90°) means that the crack propagates on the left-hand side of the graphene sheet along the direction perpendicular to the direction of the initial crack.

## 2. Results and Discussion

### 2.1. In-Plane Tearing of a Graphene Ribbon

#### 2.1.1. Effect of Loading Speed at 1 K

**Figure 2:**
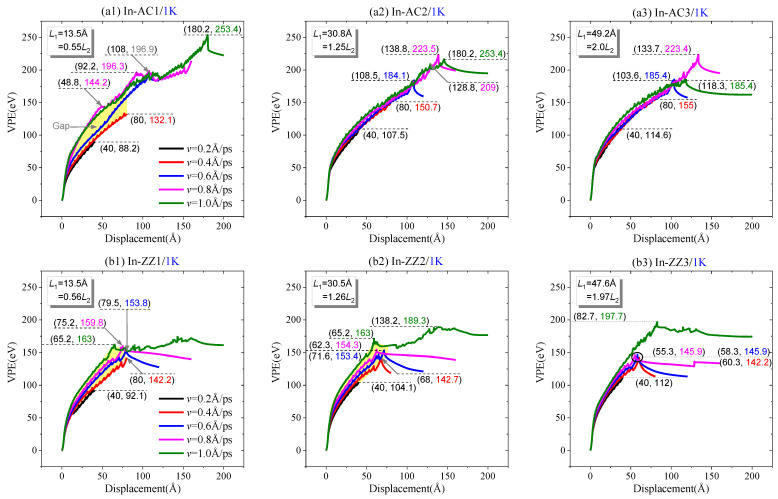
Curves of VPE vs. the tearing displacement, i.e., *d*, of a graphene sheet with three different aspect ratios at different tearing speeds within 200 ps at 1 K: (**a1**–**a3**) along the armchair crack, or (**b1**–**b3**) along the zigzag crack.

**Figure 3:**
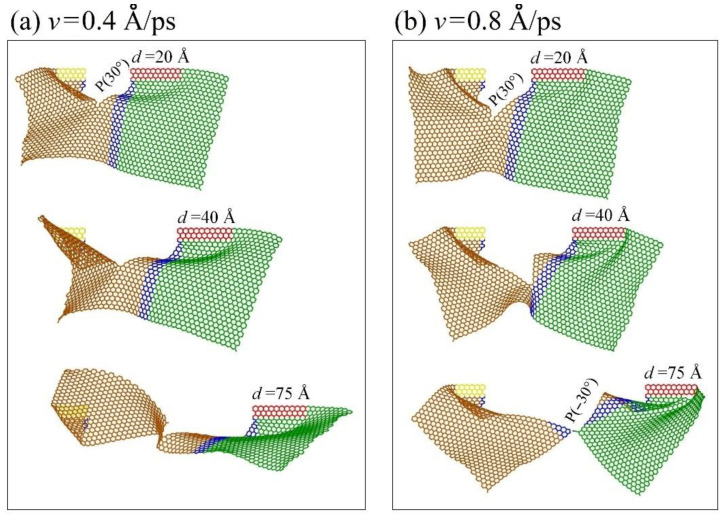
Snapshots of the graphene sheet (In-AC1 with AR = 0.55) in an in-plane tearing under different loading speeds at 1 K, e.g., (**a**) at *v* =0.4 Å/ps, (**b**) at *v* =0.8 Å/ps.

First, we studied the effect of loading speed (*v*) on the crack propagation in the graphene sheets with initial cracks as shown in Figure 1a,b. During in-plane tearing of the graphene sheet at 1 K, bond breakage takes place, and leads an increase in the potential energy of the system, which can be verified by the jagged curves shown in Figure 2. We also found that the sheet had a large deformation at both sides of the crack (Figure 3). For the *sp*^2^ carbon sheet, its bending deformation also leads to increasing potential energy, while an overlap of two parts of the sheet via van der Waals interaction reduces the potential energy of the system. For monitoring the state of the sheet during a tearing process, here we adopted a concept named variation of potential energy (VPE), which can be calculated by subtracting the initial potential energy (PE(0)) of the system from the current potential energy (PE(*t*)), i.e.,

VPE(*t*) = PE(*t*) − PE(0) (1)

Briefly, in a tearing process, VPE is determined by three factors, i.e., the number of broken C-C bonds (5~6 eV per atom [31]), the curvature of the deformed parts (with the bending stiffness of 0.1~0.4 eV/(nN·nm) [52]), and the overlap area of the deformed parts of the sheet (−0.04 eV per atom [53]).

When the graphene sheet completely breaks, VPE shows a sharp decrease, and then tends to remain constant due to the relaxation of the two pieces after they disconnect. The peak values of VPE may also be different at different loading speeds (Figure 2). For the same graphene with the same values of *L*_1_ and *L*_2_ (i.e., the lengths of the edges with confined atoms for tearing), e.g., In-AC1 (Figure 2a1), VPE increases much faster at *v* >0.4 Å/ps than those at the lower loading cases. When *v* = 0.2 and 0.4 Å/ps, VPE continues increasing monotonously. Since they have the same loading duration, i.e., 200 ps, the sheet does not break after 200 ps of tearing. However, as *v* ≥ 0.6 Å/ps, VPE shows a sharp decrease, indicating that the sheet breaks. Moreover, the peak value of VPE is higher at a greater loading speed.

Before breakage of the graphene sheet, the VPE curves are not well aligned at different loading speeds (see the gap marked in Figure 2a1). For example, the VPE curves with respect to *v* = 0.4 and 0.8 Å/ps have a large gap. By comparing the snapshots in Figure 3a,b, the path of crack propagation is along P(30°) at *v* = 0.4 Å/ps, while the path at *v* = 0.8 Å/ps is along P(30°) at first, and then changes to P(−30°) (Supplementary Video S1). Hence, the gap between the two corresponding VPE curves is mainly caused by the different paths of crack propagation at different loading speeds.

When more atoms are confined in tearing, e.g., *L*_1_ = 30.8 Å (In-AC2 in Figure 2a2) or *L*_1_ = 49.2 Å (In-AC3 in Figure 2a3), the gaps between the VPE curves are much smaller than those in In-AC1 (Figure 2a1). This implies that the loading speed has a slight influence on the path of crack propagation when more atoms at the edge are confined in tearing. However, the peak value of VPE is generally higher at faster loading speeds.

We also evaluated the cracking propagation on the sheet with the initial zigzag crack; Figure 2b1 indicates that, when fewer atoms are confined, the VPE curves have obvious gaps during loading at different speeds. Meanwhile, the peak values of VPE have slight differences. Similar characteristics can be found in the curves in Figure 2b2. The reason for this is that the sheet has greater deformation when torn at a higher speed. However, in Figure 2b3, the gaps between the VPE curves are small, which indicates that the sheet has the same path of crack propagation (P(0°)) before the crack tip approaches the lower edge of the sheet. However, the VPE curve of the sheet at *v* = 1.0 Å/ps has a peak value much higher than those at lower loading speeds, because the sheet has extra broken bonds that occur after the crack tip approaches the lower edge of the sheet.

We also found that the peak values of VPE in Figure 2b were close to 150 eV, which is about 40 eV less than those in Figure 2a. This is because the sheets have different numbers of broken bonds occurring on different paths of the crack propagation, e.g., the armchair crack extends along with P(30°) or P(−30°), while the zigzag crack extends forward (P(0°)).

#### 2.1.2. Effect of Aspect Ratio at 1 K

**Figure 4:**
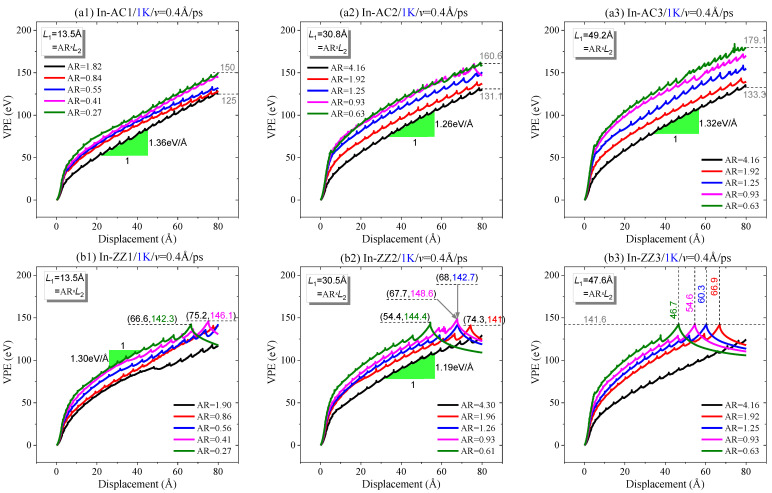
Curves of VPE vs. the in-plane tearing displacement of graphene ribbons with *v* = 0.4 Å/ps and different aspect ratios at 1 K: (**a1**–**a3**) an armchair crack in the sheet, (**b1**–**b3**) a zigzag crack in the sheet.

**Figure 5:**
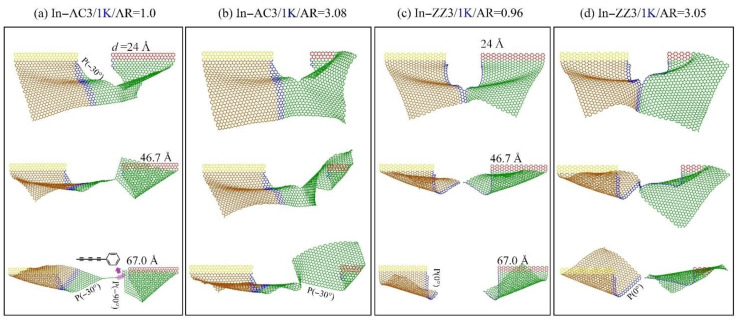
Snapshots of the sheets in an in-plane tearing with *v* = 0.4 Å/ps at 1 K: (**a**,**b**) In-AC3 with AR = 1.00 or 3.08, respectively; (**c**,**d**) in-ZZ3 with AR = 0.96 or 3.05, respectively.

From Figure 4, it can be seen that each VPE curve has a sharp increase at the initial stage. Its slope decreases gradually and tends to be constant (Figure 4a). When the moving area contains more atoms, a longer displacement of the moving area is required for a VPE curve to reach the constant slope. This is due to two reasons: One is that the bond breakage starting from the crack tip leads to a sharp increase in VPE, while the bending deformation of the sheet causes a slower increase in potential energy (Figure 5). Due to different numbers of confined atoms in the moving area, the other reason is that the sheet has different deformation due to the local displacement constraint on the atoms near the two confined areas. Hence, the slope tends to be constant when the crack expands deeply, which requires a longer tearing displacement of the moving area. We also estimated the slopes of the curves in different cases. According to the data shown in Figure 4a, the slope was around 1.3 eV/Å for the three In-AC cases.

When tearing a sheet along the zigzag crack (Figure 4b), the VPE curves have different slopes when the same sheet has different lengths of the moving area (*L*_2_ or AR) before the sheet breaks in half. When observing the cracking processes, we found that the crack always propagated along with the path P(0°) perpendicular to the loading direction (Figure 5c,d). Hence, the gaps between the VPE curves must be caused by the different deformation of the sheets with different values of AR at the same tearing speed.

To show the influence of AR on the path of crack propagation, the paths are listed in Table 2. One can see that when the sheet has an armchair crack, the crack propagates along with the path P(30°) or P(−30°), depending on the value of AR. Note that the two paths produce a smooth edge in a zigzag direction. However, when tearing the sheet with a zigzag crack, the propagation path is mainly along P(0°). This implies that the crack is liable to propagate along a zigzag direction.

**Figure 6:**
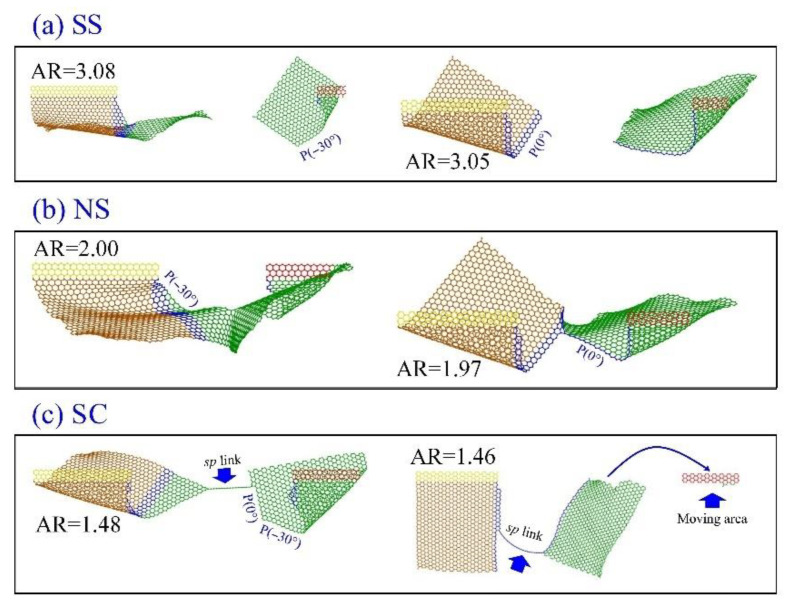
Propagation styles of the cracks in sheets under an in-plane tearing at 1 K: (**a**) “SS” for split with smooth edges at *v* = 0.6 Å/ps, (**b**) “NS” for not split off when *v* = 0.2 Å/ps, and (**c**) “SC” for split but still connected via an *sp* carbon link when *v* = 1.0 Å/ps. In each of the three panels, the left-hand sheet is the In-AC3 and the right-hand one is the In-ZZ3.

To show the propagation of the crack in a sheet, herein, the three typical styles of propagation are demonstrated. As shown in Figure 6a, the graphene sheet splits off with new smooth edges. In this case, the crack propagation style is marked as “SS”, i.e., smoothly split. In Figure 6b, the sheet does not split off, and is marked “NS” as the propagation style. Sometimes, the path of crack propagation may vary during tearing. For example, in Figure 6c, the path first propagates along P(−30°), and then along P(0°). Meanwhile, the two parts of the sheet are connected via an *sp* carbon link. In this case, the style is marked as “SC”. Sometimes, the moving area on the sheet may depart when the crack propagates in the “SC” style. A similar phenomenon has been mentioned in the work by Omeltchenko et al. [42], i.e., the second branches and the overhangs appear in the process of crack propagation.

Table 3 lists the propagation styles of the sheets with an initial armchair or zigzag crack. It illustrates that the sheet does not split off (“NS” in Table 3) when the tearing speed is low, e.g., 0.2 Å/ps with 200 ps. When a higher speed is applied to the atoms in a small moving area (e.g., AR_1_), the sheet still does not split off. For the sheet with an armchair crack, its path of crack propagation is mainly in the “SC” style. Conversely, the propagation path of a zigzag crack is mainly split off, with smooth edges along the initial direction of the crack, which benefits the graphene patterning by tearing.

#### 2.1.3. Effects of Temperature on In-Plane Tearing

**Figure 7:**
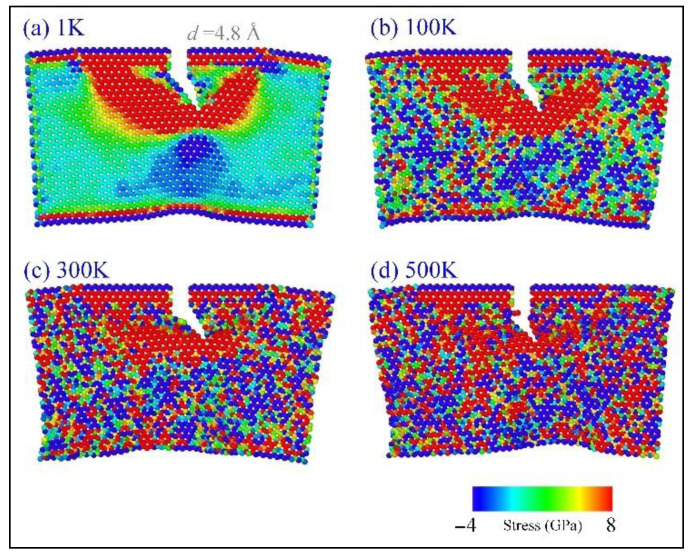
The virial stress distribution in a graphene sheet with an armchair crack in tearing (with *v* = 0.6 Å/ps and AR = 1.25) at (**a**) 1 K, (**b**) 100 K, (**c**) 300 K, or (**d**) 500 K.

**Figure 8:**
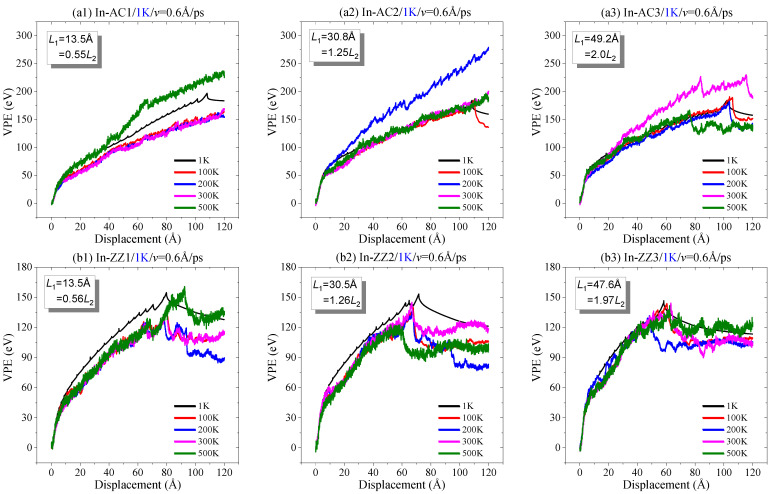
Curves of VPE vs. the in-plane tearing displacement of graphene sheets with *v* = 0.6 Å/ps at different temperatures: (**a1**–**a3**) an armchair crack in the sheet, (**b1**–**b3**) a zigzag crack in the sheet.

In the above discussion, the dynamic response of the crack in torn graphene is collected at an extremely low temperature, i.e., 1 K, which is difficult, but can be achieved in an experiment. It is known that the ambient temperature of the graphene sheet indicates the intensity of the thermal vibration of atoms. In general, a stronger thermal vibration of atoms leads to a lower strength of the sheet (Figure 7). At 1 K, the thermal vibration has slight influence on the bond length and bond angles in tearing. In other words, the crack propagation simply depends on the essential loading factors, e.g., the loading speed and the value of AR. One fact that we have to face is that graphene tearing could happen at higher temperatures. Consequently, the sheet may behave differently when being torn at different temperatures. Hence, the VPE curve at a higher temperature (Figure 8) is thicker due to stronger thermal vibration that induces a larger fluctuation of the potential energy of the system; Figure 8 indicates that the VPE curves at higher temperatures are not always aligned with that at 1 K. For instance, the VPE curves at higher temperatures are not as smooth as that at 1 K; meanwhile, they may have obvious gaps.

**Figure 9:**
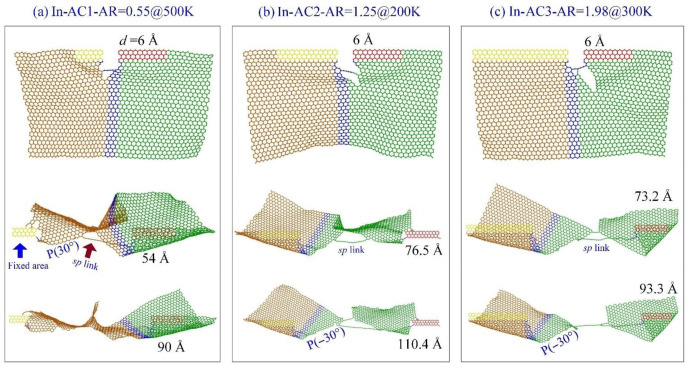
Snapshots of graphene sheets with different values of AR in an in-plane tearing with *v* = 0.6 Å/ps at different temperatures, e.g., (**a**) In-AC1 at 500 K, (**b**) In-AC2 at 200 K, (**c**) In-AC3 at 300 K.

When tearing along the armchair crack (Figure 8a), some of the curves are aligned very well before the graphene sheet breaks. For example, the curves in In-AC1 have a small gap when the temperature is between 100 K and 300 K. In the In-AC2 case, the curves (except that at 200 K) have small gaps. In the In-AC3 case, only the curve at 300 K has an obvious gap with the remaining curves. By observing the snapshots of the sheet in the three cases (Figure 9), we found that the crack mainly propagated along with P(30°) or P(−30°), which was the same as happened at 1 K. Moreover, the sheet had a large deformation in tearing. However, there an *sp* carbon link appeared at the crack edge (i.e., propagating in an “SC” style), which was obviously different from that at 1 K. The reason for this is that the stress distributes randomly in the sheet at higher temperatures (Figure 7). This kind of deficient propagation should be avoided in graphene patterning.

When tearing the sheet with a zigzag crack, the crack propagates in the “SS” style. Each of the VPE curves in Figure 8b has at least a sharp decrease. The peak values have slight differences, indicating that the breakage of the sheet slightly depends on the ambient temperature. After the sharp decrease, the curves still have large fluctuation due to the large continuous deformation of the sheet.

In Figure 9, the origin of an *sp* link should be explained; Figure 7 indicates that the atoms near the crack tip have higher stresses. In the area in front of the crack tip, new bond breakage may happen randomly due to the thermal vibration of the atoms in the area. When the broken bond is not right at the crack tip, a nanopore is produced, and the crack tip jumps into the pore and begins further propagation. This phenomenon is extremely remarkable when discovered in an MD simulation. When the crack tip moves forward, the atoms between the two crack edges will be under tension at a high stress level. Hence, bonds break until forming an sp link, which has a higher tensile strength than an *sp*^2^ C-C bond.

### 2.2. Out-of-Plane Tearing of Graphene Ribbons

**Figure 10:**
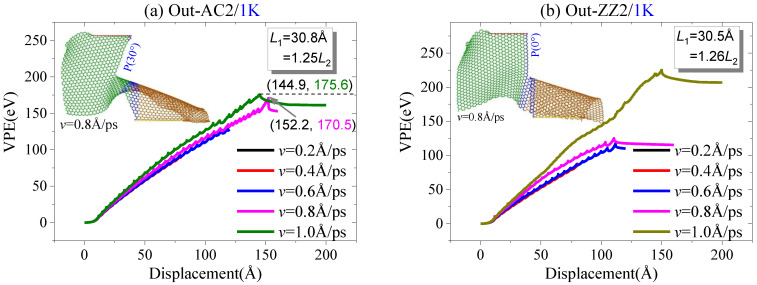
Curves of VPE vs. out-of-plane tearing of graphene ribbons with three aspect ratios at different speeds at 1 K: (**a**) armchair crack; (**b**) zigzag crack.

In an out-of-plane tearing process, the moving area has a velocity perpendicular to the sheet, i.e., *v* = *v*_z_. First, the effect of the tearing speed on the crack propagation is evaluated. According to the molecular dynamics simulation results, e.g., as shown in Figure 10, the loading speed has a slight influence on the crack propagation. For example, the armchair crack on the Out-AC2 expands along with P(30°) when AR = 1.25 (Figure 10a). The zigzag crack propagates along with P(0°) when AR = 1.26 (Figure 10b). If the tearing speed is too high, e.g., *v* = 1.0 Å/ps, the moving area may escape from the sheet, which leads to an increase in VPE, since more broken bonds occur at the edges of the moving area.

**Figure 11:**
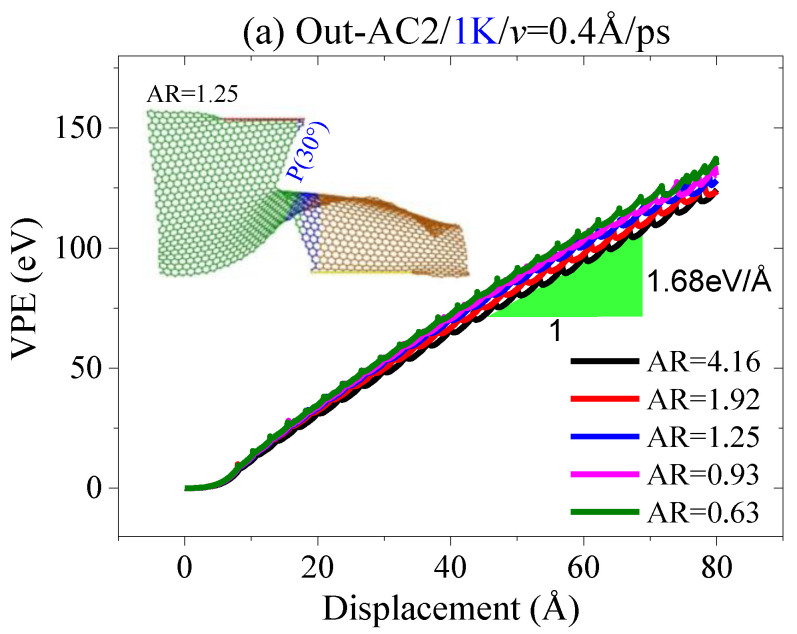

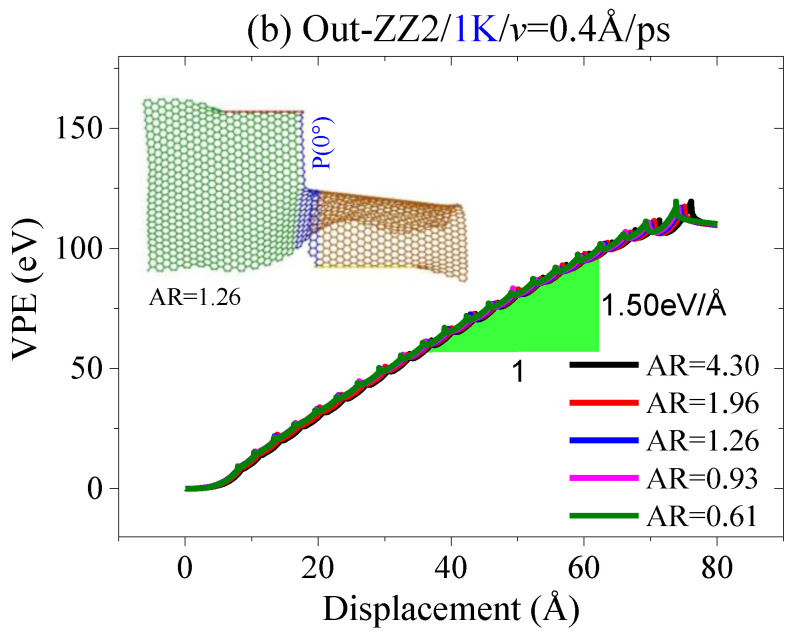
Curves of VPE out-of-plane tearing with the speed of *v* = 0.4 Å/ps at different aspect ratios and at 1 K: (**a**) armchair crack; (**b**) zigzag crack.

Second, the value of AR also has a slight influence on the path of crack propagation; Figure 11a indicates that the VPE curves have a slight gap and the slopes are about 1.68 eV/Å when tearing the sheet along the armchair crack, which expands along with the path of P(30°). When tearing the zigzag crack, the path of crack propagation is along P(0°). The inserted snapshots in Figure 11 demonstrate that the deformation of the sheet is very low in the out-of-plane tearing. Hence, stress concentration at the crack tip can be avoided. This is the reason why the curves in Figure 11 have slight gaps while the corresponding VPE curves in Figure 4 have obvious gaps, meaning that the lengths of the two confined areas in the graphene sheet can be set randomly in an out-of-plane tearing.

**Figure 12:**
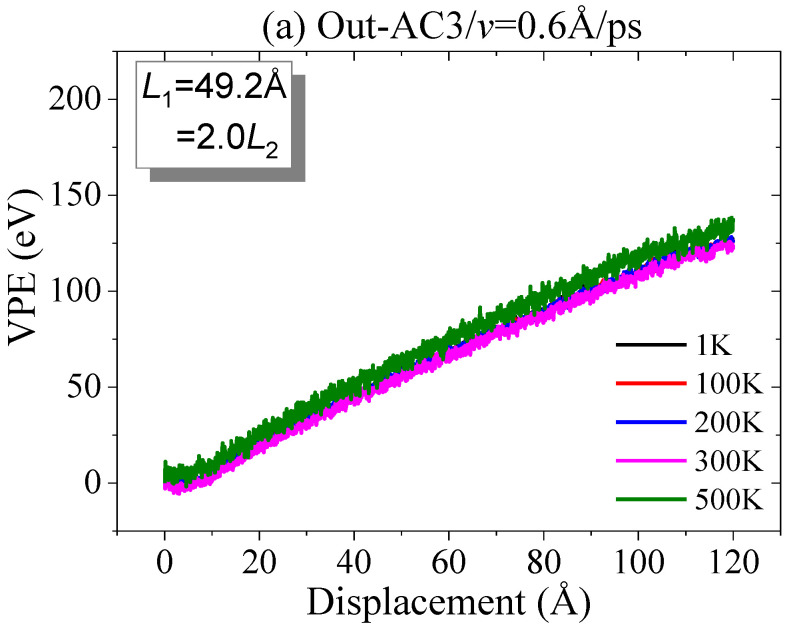

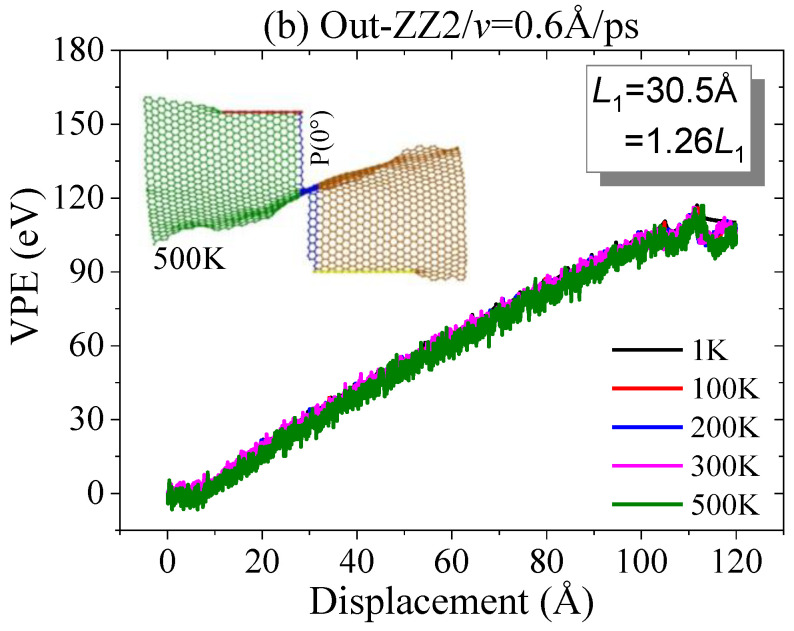
Curves of VPE vs. out-of-plane tearing of graphene ribbons with three aspect ratios at different temperatures: (**a**) armchair crack; (**b**) zigzag crack.

Finally, we investigated the effects of temperature on the crack propagation of the crack in the graphene sheet torn out-of-plane. The results in Figure 12 illustrate that the thermal vibration of atoms has no influence on the path of crack propagation when the graphene sheet is torn in a thermostat with a temperature no higher than 500K. It can be concluded that the out-of-plane tearing will benefit the patterning of graphene sheets, because the effect of temperature is negligible.

### 2.3. Oblique Tearing

**Figure 13:**
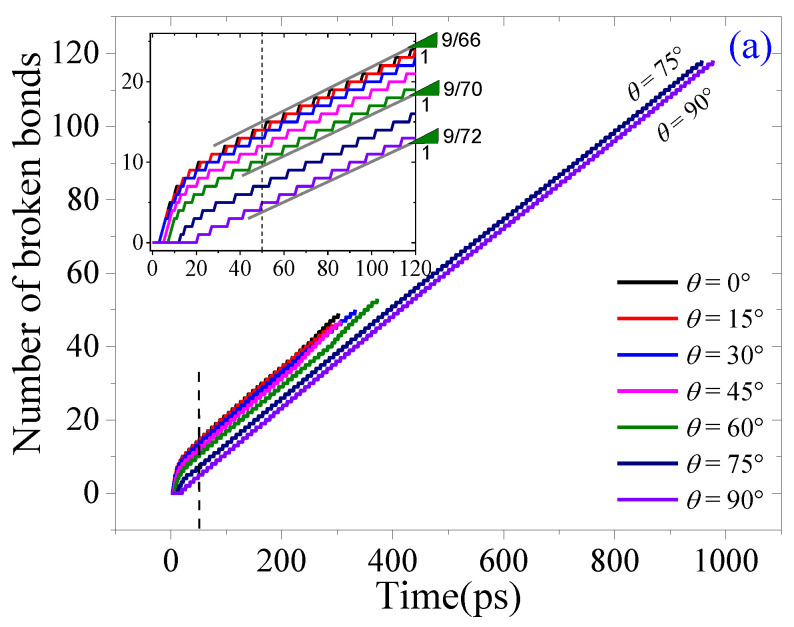

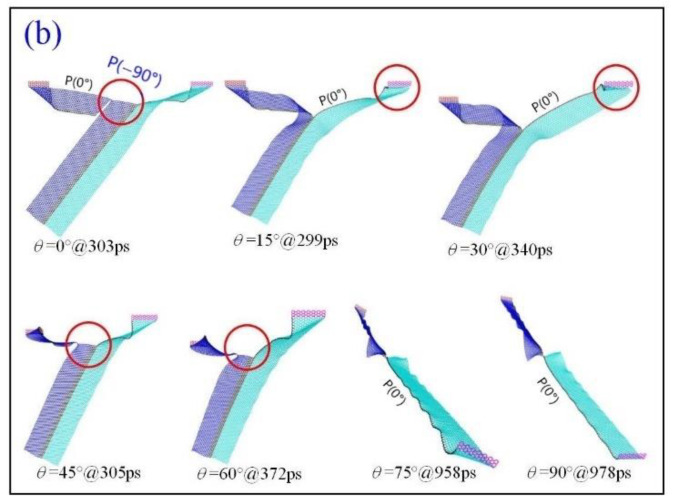
Results of a graphene sheet in oblique tearing conditions with *v* = 0.6 Å/ps at 1 K: (**a**) numbers of broken bonds, (**b**) the snapshots of the sheet torn in different directions.

According to the tearing direction shown in Figure 1e, oblique tearing happens when *θ* is between 0° and 90°. Herein, a sheet with a size of ~58 Å × 301 Å was selected, with an armchair crack at the center of the zigzag edge (~58 Å). The top five rows of atoms at the zigzag edge were confined as the fixed area or the moving area for applying the tearing load. Five cases of oblique tearing together with the in-plane and out-of-plane tearing results were collected for comparison. For example, in Figure 13a, the VPE curves have different slopes at the initial stage, and then tend to be constant after about 50 ps. Their constant slopes are slightly different.

There are three types of tearing results. For example, when *θ* =75° or 90°, the crack always propagates along with the path P(0°) (Figure 13b). When *θ* < 75°, the path is first aligned with P(0°) for about 300 ps, and then changes to be aligned with P(−90°) (see the snapshots with *θ* = 0°, 45°, or 60° in Figure 13b), or a new crack appears at the end of the moving area (see the snapshots with *θ* = 15°, or 30° in Figure 13b). The sheet splits off by generating different numbers of broken bonds (Figure 13a). Hence, even when the sheet has a zigzag crack, the path of crack propagation predominantly depends on the loading direction. Note that a rectangular ribbon can be produced from a wider graphene sheet by the oblique tearing processes, where the crack path is along P(0°) at first, and then turns along P(−90°).

## 3. Methodology

In this study, the tearing process of graphene sheets is simulated using the molecular dynamics approach, and conducted on the open-source code LAMMPS [54]. Each simulation contains the following main steps:

Step 1: Build the graphene sheet with an initial crack;

Step 2: Reshape the sheet by minimizing its potential energy;

Step 3: Before tearing, relax the system in an NVT ensemble for 100 ps, using the Nose–Hoover thermostat [55,56] to control temperature;

Step 4: Fix the atoms in the fixed area, and apply a constant speed on the atoms in the moving area along a direction for 200 ps;

Step 5: Collect the essential data for post-processing.

In a simulation, the interactions between the carbon atoms in the graphene sheet are determined by the adaptive intermolecular reactive bond-order (AIREBO) potential [57], with a cutoff distance of 2 Å for the bonding interaction and 10 Å for the non-bonding interaction. The time step is set to be 0.001 ps throughout the entire process. The virial stresses [58] of atoms in the simulations are predicted with 3.4 Å as the thickness of the sheet.

## 4. Conclusions

In this study, we estimated the fracture properties of pre-cracked graphene during the tearing process with constant velocity. The effects of the aspect ratio, loading speed, loading direction, and ambient temperatures on the crack propagation in the monolayer graphene sheet were investigated using the molecular dynamics simulation approach. Some remarkable conclusions can be drawn based on the numerical results for preparing a graphene ribbon by tearing; for example:

Firstly, in an in-plane tearing process at an extremely low temperature, a high loading speed on the graphene sheet with an initial armchair crack may produce a complicated path of crack propagation. The influence can be reduced when the two loading areas (i.e., the fixed area and the moving area) in the sheet contain more atoms. If the sheet has an initial zigzag crack, the crack propagates straight forward, i.e., along P(0°).

Secondly, when the two loading areas contain fewer atoms, a new crack may occur at the edge of the shorter area—especially at a higher loading speed, e.g., >0.4 Å/ps. An *sp* carbon link may appear soon after the initial crack propagates through the whole graphene sheet. These appear more frequently in the graphene sheets with armchair cracks than in the sheets with zigzag cracks.

Thirdly, in an in-plane tearing of a graphene sheet with an initial armchair crack, temperature influences the crack propagation. For example, at a temperature higher than 200 K, an *sp* carbon link may appear on the path of crack propagation, i.e., the crack tip passes through the carbon link due to a slight stress difference near the crack tip when the atoms have strong thermal vibration. Fortunately, the *sp* carbon link seldom appears in the graphenes sheet with initial zigzag cracks.

Fourthly, in an out-of-plane tearing process, if the loading speed is less than 1 Å/ps, the armchair crack propagates along P(30°), while the zigzag crack propagates along P(0°), despite the length difference between the two loading areas and the difference in ambient temperature. Hence, out-of-plane tearing is more suitable for graphene patterning than in-plane tearing.

Finally, when an oblique tearing process is applied to a larger sheet with an initial zigzag crack, the path of propagation depends on the loading direction.

From above, for preparing a graphene ribbon from a sheet by tearing, the *sp* carbon links on the path of the armchair crack propagation should be avoided, and the two loading areas should have sufficient length to counteract the local stress concentration.

## Figures and Tables

**Table 1:** Initial parameters of graphene ribbons with different aspect ratios (AR).

Model	*L*_1_ (Å) = AR × *L*_2_	*L*_2_ (Å)	AR = *L*_1_/*L*_2_
			AR_1_/AR_2_/AR_3_/AR_4_/AR_5_
AC1	13.5	7.4/16.0/24.6/33.2/49.2	1.82/0.84/0.55/0.41/0.27
AC2	30.8		4.16/1.92/1.25/0.93/0.63
AC3	49.2		6.65/3.08/2.00/1.48/1.00
ZZ1	13.5	7.1/15.6/24.2/32.7/49.7	1.90/0.86/0.56/0.41/0.27
ZZ2	30.5		4.30/1.96/1.26/0.93/0.61
ZZ3	47.6		6.70/3.05/1.97/1.46/0.96

**Table 2:** Crack propagation paths in the in-plane tearing of graphene sheets with *v* = 0.4 Å/ps at 1 K.

Model	AR_1_	AR_2_	AR_3_	AR_4_	AR_5_
In-AC1	P(−30°)	P(30°)	P(30°)	P(30°)	P(30°)
In-AC2	P(−30°)	P(−30°)	P(−30°)	P(−30°)	P(30°)
In-AC3	P(−30°)	P(−30°)	P(−30°)	P(30°)	P(−30°)
In-ZZ1	P(0°)	P(0°)	P(0°)	P(0°)	P(0°)
In-ZZ2	P(0°)	P(0°)	P(0°)	P(0°)	P(0°)
In-ZZ3	P(−30°)	P(0°)	P(0°)	P(0°)	P(0°)

Note that the same crack paths share the same background.

**Table 3:** Crack propagation styles of the armchair and zigzag cracks in graphene sheets at 1 K.

	In-AC3					In-ZZ3				
	AR_1_	AR_2_	AR_3_	AR_4_	AR_5_	AR_1_	AR_2_	AR_3_	AR_4_	AR_5_
*v* = 0.2	NS	NS	NS	NS	NS	NS	NS	NS	NS	NS
*v* = 0.4	NS	SC	SC	SC	SC	NS	SS	SS	SS	SS
*v* = 0.6	SC	SS	SS	SS	SC	SC	SS	SS	SS	SS
*v* = 0.8	SS	SS	SS	SC	SC	SS	SS	SS	SS	SS
*v* = 1.0	SC	SC	SC	SC	SC	SC	SS	SS	SC	SC

Note that the same crack styles own the same background.

## Data Availability

This paper does not report any data.

## Supplementary Materials

The supporting information can be downloaded at: https://www.mdpi.com/article/10.3390/ijms23126471/s1.
